# Efficient and Sustainable Electrochemical Demolition of Hard‐Metal Scrap with Co‐Rich Binder

**DOI:** 10.1002/cssc.202402218

**Published:** 2025-03-17

**Authors:** Francesco Tavola, Gian Pietro De Gaudenzi, Giulio Bidinotto, Francesco Casamichiela, Andrea Pola, Sandra Tedeschi, Benedetto Bozzini

**Affiliations:** ^1^ Dipartimento di Energia Politecnico di Milano via Lambruschini 4 20156 Milano Italy; ^2^ O.M.C.D. Tek Hub Via Megolo, 49 I-28877 Anzola d'Ossola VB) Italy

**Keywords:** hardmetal, critical raw materials, corrosion, cobalt, tungsten

## Abstract

Cobalt and tungsten hold strategic importance in various industries, and fostering their circular economy could lead to cost reduction and a more sustainable use of natural resources. Eco‐ friendly electrochemical recovering processes are promising alternatives to the state‐of‐the‐art pyrometallurgical approaches, but their productivity is too low for industrial standards. Low demolition rates are caused by hardmetal pseudopassivation phenomena. In previous work, we demonstrated that a pulsed‐potential approach, employing a neutral aqueous solution and alternating pseudopassivating film formation with its mechanical removal by oxygen evolution reaction, thus refreshing an active HM surface, is effective in avoiding the corrosion‐self‐termination for corrosion‐resistant grades. This study extends this approach to the most widespread grades, featuring Co‐rich binders. This new application required fine‐tuning of the operating conditions to adapt them to the target grades. Electrochemical characterization of the psudopassive film growth in this study is centered on cyclic voltammetry and potentiostatic polarization. Corroded hardmetal and detached pseudopassive films were subjected to morphological and compositional analyses with scanning electron microscopy and x‐ray fluorescence mapping. We thus demonstrated that optimized pulsed anodic potentiostatic polarization enables efficient demolition of hard metal coupons, combined with separation of Co and W, at high rate and with low energy consumption.

## Graphical abstract

Cobalt and tungsten are vital in various industries, and enhancing their circular economy can reduce costs and promote sustainability. An eco‐friendly electrochemical recovery method using pulsed‐potential and OER effectively overcomes pseudopassivation issues in hardmetals. This study adapts the approach for Co‐rich binders, demonstrating high demolition throughput and efficient separation of Co and W with optimized pulsed anodic polarization conditions.

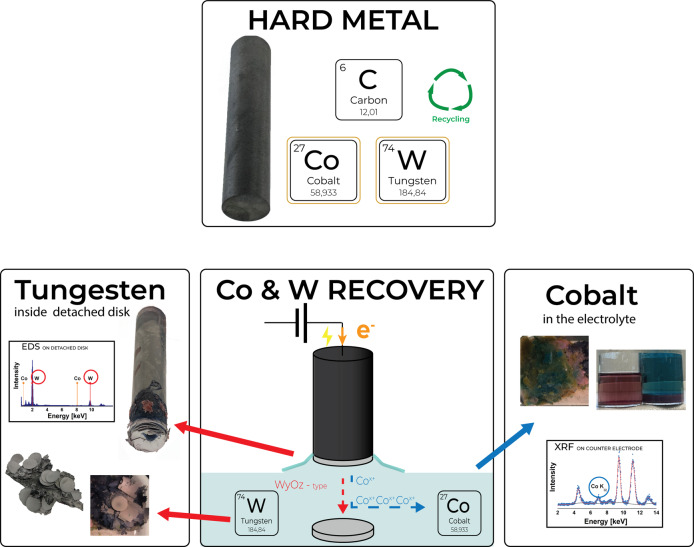

## 1 Introduction

Hardmetal (HM) is a sintered material composed of tungsten carbide (WC) particles embedded in a cobalt binder. Hardness of WC, combined with ductility of Co make HM an ideal solution for high‐wear components subjected to harsh mechanical stresses.[^1^, ^2^, ^3^, ^4^] A pressing aspect of widespread HM usage is that tungsten and cobalt are Critical and Strategic Raw Materials (CRM, SRM),^[5]^ making scrap recycling mandatory. Moreover, nowadays cobalt resources are put under additional stress by the increasing growth of automotive battery demand.^[6]^ State‐of‐the‐art HM recycling relies on chemical approaches or the pyro‐metallurgical zinc process.^[7]^ In the former, a complex sequence of pyro‐ and hydrometallurgical treatments based on the use of mineral acids and/or alkaline solutions is implemented to fully dissolve scraps. In the latter method HM scrap is pulverized benefiting of the high solubility of zinc in cobalt and the attending substantial lattice parameters variation. As a consequence, usage of aggressive chemicals with high environmental impact and a large energy treatment step of the recovery process.[^8^, ^9^] For about three decades, electrochemical demolition has been attempted to minimize the environmental impact and to shift towards green recovering technologies. Specifically, an HM coupon, polarized anodically, can be corroded and dissolved using appropriate electrolytes and electrochemical conditions. After the dissolution step, Co or Co(II) oxy‐hydroxides and basic salts can be electrodeposited at the counter‐electrode, that can be graphite or some metal that is stable in the selected electrolyte.[^8^, ^10^] The fate of W strongly depends on the electrolyte and the electrochemical polarization. In fact, acidic and neutral environments favor Co dissolution and formation of Co(II) colloids, while alkaline electrolytes also enable tungsten dissolution.[^2^, ^4^] It is worth noting that in the literature, with the exception of [9], only acidic and alkaline electrolytes have been considered, that make the process less ecologically attractive. A crucial common aspect to all electrochemical scrap treatment routes is the pseudo‐passivation behavior of HM that inhibits continuous material extraction.^[11]^ In fact, with the formation of W‐enriched chemically and electrically insulating porous corrosion product layers, resulting from surface Co depletion, the corrosion process is never totally impeded, but it is drastically slowed.^[11]^ While in the literature, attempts to get rid of pseudopassivation have been centered on the use of chemical additives and mechanical intermediate steps, our research group has recently proposed an efficient and simple solution to break the strongly W‐enriched inhibition layer with the mechanical action resulting from the application of an Oxygen Evolution Reaction (OER) period, alternated with periods of pseudopassive film formation.^[9]^ It is fair to recall here, that during the review process of our original submission – based on a MSc thesis published on the web in April 2022^[12]^ – that started in October 2022 and was completed only at the end of July 2023, a paper appeared^[13]^ in April 2023 proposing a very similar approach, that can be regarded as simultaneous discovery.

The aim of the present paper is to report on upgrade and fine‐tuning of this approach, originally discovered for highly corrosion‐resistant HMs,^[9]^ to the case of grades with unalloyed Co binder. It is worth stressing that this kind of HM is by far the most industrially employed one, generating the largest proportion of scrap, that is scantily recycled owing to its unfavourable Co/W proportion. In addition, we also focused on defining operating conditions that would jointly optimize productivity, Co−W separation and energy efficiency.

## 2 Results and Discussion

Our study is organized as follows. (i) Electrochemical characterization of the corrosion of the grade considered: Co12WC88. This corrosion study is aimed at defining the parameters of PS square‐wave cycling demolition loops (PCDL). (ii) Analyses of the corroded material, to assessing the nature of the pseudopassive films and the efficiency of Co and W separation. (iii) Tuning of the electrical variables of the electrochemical demolition process in view of Energetic optimization. Cyclic voltammetry (CV) and potentiostatic (PS) measurements were used to investigate sample corrosion. CV monitored variations in anodic voltage and the development of irreversible processes. PS tests allowed to assess the onset of the OER. Following our previous work,^[9]^ PCDL based on periodic PS switching between pseudopassive film growth conditions (E_critic,PP_) and OER (E_critic,OER_) for different time intervals t_pp_ and t_OER_ were applied. More details on materials characterization technique and electrochemical protocols are available in Section 4.

### 2.1 Electrochemical Characterization of the Co12WC88 HM Grade and Definition of PCDL Parameters

**Figure 1:**
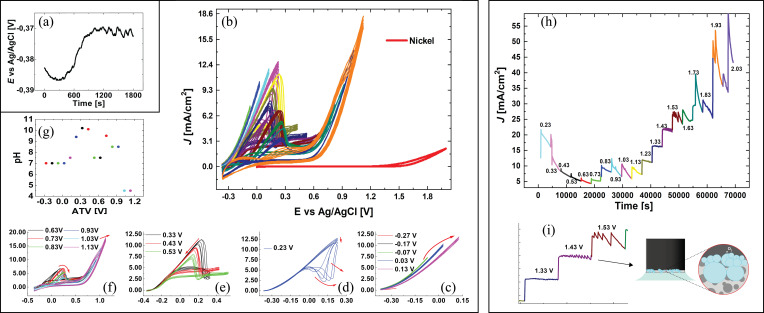
Electrochemical characterization of the 12%Co‐WC HM grade in 0.2 M Na_2_SO_4_ aqueous solution. (a) Open‐circuit potential (OCP) measurement; (b‐f) CVs with progressively increasing anodic terminal voltage (ATV). (b) overview of all CVs on HM samples and Ni foil (red plot). (c‐f) CVs grouped according to classes of corrosion behaviour: (c) active corrosion; (d) incipient pseudopassivation; (e) fully developed pseudopassivity; (f) transpassivity. (g) pH at the end of CV at indicated ATVsattained by the electrolyte at the end of each CV . (h) Potentiostatic staircase (PSS) tests, highlighting the development of the oxygen evolution reaction (OER) (i).

As hinted at in the Introduction, the anodic behaviour of HM is strongly irreversible and it depends on the grade composition, aggressive ambient chemistry and on [9]. With materials exhibiting such complex electrochemical behaviour, CV with progressive changes of terminal voltages is a powerful tool for corrosion characterization. In fact, the repeated application of linear voltammetric scans, with appropriate anodic and cathodic terminal voltages, is able to induce and monitor irreversible modifications of the surface of both the binder and hard phase, corresponding to active corrosion, pseudopassivation and transpassivity. Open circuit potential (OCP) measurements, carried out prior to CVs, show an initial transient after which a steady‐state value of −0.37 V±0.001 vs. Ag/AgCl is attained, typical of Co immersion potential (Figure 1a). The whole CV series, reported in Figure 1b, clearly shows, on the one hand, active corrosion, pseudopassive and transpassive behaviours and, on the other hand, irreversibilities brought about by increasing applied anodic polarization. The CV measured in the same electrolyte at a Ni electrode shows that the whole scanned anodic terminal potential (ATV) range is below the OER threshold, demonstrating that – at the highest applied anodic potentials –, the quasi‐Tafel increase in current density (c.d.) is due to transpassive HM corrosion. Panels c‐f of Figure 1 classify the kinds of corrosion behaviour found by progressively increasing the ATV. In correspondence of each class, a critical potential (*E_crit_)* can be assigned. (i) Figure 1c shows the active corrosion region of the binder, characterized by a Butler‐Volmer type c.d. growth. Nevertheless, the progressive c.d. decrease observed upon cycling denotes W enrichment of the binder, due to selective Co dissolution. (ii) Figure 1d marks the transition between active corrosion and pseudopassivation, characterized by the progressive development of a negative‐resistance region. In previous investigations, we have proved that this behaviour is due to the formation of mixed Co−W oxides.[^14^, ^15^, ^16^] (iii) Figure 1e reports CVs representative of the fully pseudopassivated state: increasing the potential, the c.d. reaches a maximum, after which it drops, reaching an asymptote that is similar to that of passivating metals, but with notably higher c.d. values, hence the denomination of pseudopassive state. In fact, pseudopassivation, corresponds to the formation of a layer that indeed inhibits corrosion, but does not stop it.^[17]^ Thus, an ATV of 0.33 V clearly separates the active dissolution behaviour from the pseudopassive one. At anodic potentials higher than this threshold, fully transpassivity develops (Figure 1f), resulting from the oxidative failure of the pseudopassive layer, chielfy by WC corrosion.[^14^, ^18^] The best potential choice for pseudopassive film formation is thus 0.23 V, where progressive W enrichment brings about the formation of a thick and porous, Co‐depleted layer. The pH attained by the electrolyte at the end of each CV measurement is reported in Figure 1g.

In order to select the correct potential for OER evolution required for electrochemical demolition,^[9]^ we employed the PSS approach (Panels h, i of Figure 1). Monitoring the c.d. time‐series in response to each PS step, OER inception can be clearly ascertained from c.d. oscillations.[^19^, ^20^] We found that no oxygen formation appears until 1.33 V, while at 1.53 V large c.d. relaxation oscillations are observed, indicating periodic bubble growth and release at the anode surface. In line with the procedure established in [9], the optimal choice is 1.43 V because in these conditions the O_2_ bubble formation rate is enough to yield film detachment without giving rise to an excessive coverage of the horizontal anode. The characteristic HM corrosion voltages, derived from CV and PSS measurements, are summarized in Table 1.

**Table 1:** Characteristic potential for HM corrosion, derived from electrochemical measurements. Active corrosion at 0.33 V and OER at 1.43 V were selected for the low‐ and high‐potential steps for electrochemical demolition.

Characteristic processes potentials	Ecrit [V]
OCP – quasi stable conditions	−0.37
Active corrosion	0.23
Incipient pseudopassivation	**0.33**
Fully developed pseudopassivation	0.43
Fuly developed transpassivity	0.63
OER	1.43

### 2.2 The PCDL Process for HM Grades with Unalloyed Co Binder

#### 2.2.1 Electrochemical Measurements

In our previous study on highly corrosion‐resistant grades,^[9]^ we demonstrated the superiority of the PCDL method over potentiostatic and galvanostatic polarization modes for the demolition of HM scrap, in terms of: (i) productivity, (ii) capability of separating Co and W during the demolition step itself and (iii) energy demand minimization.

**Figure 2:**
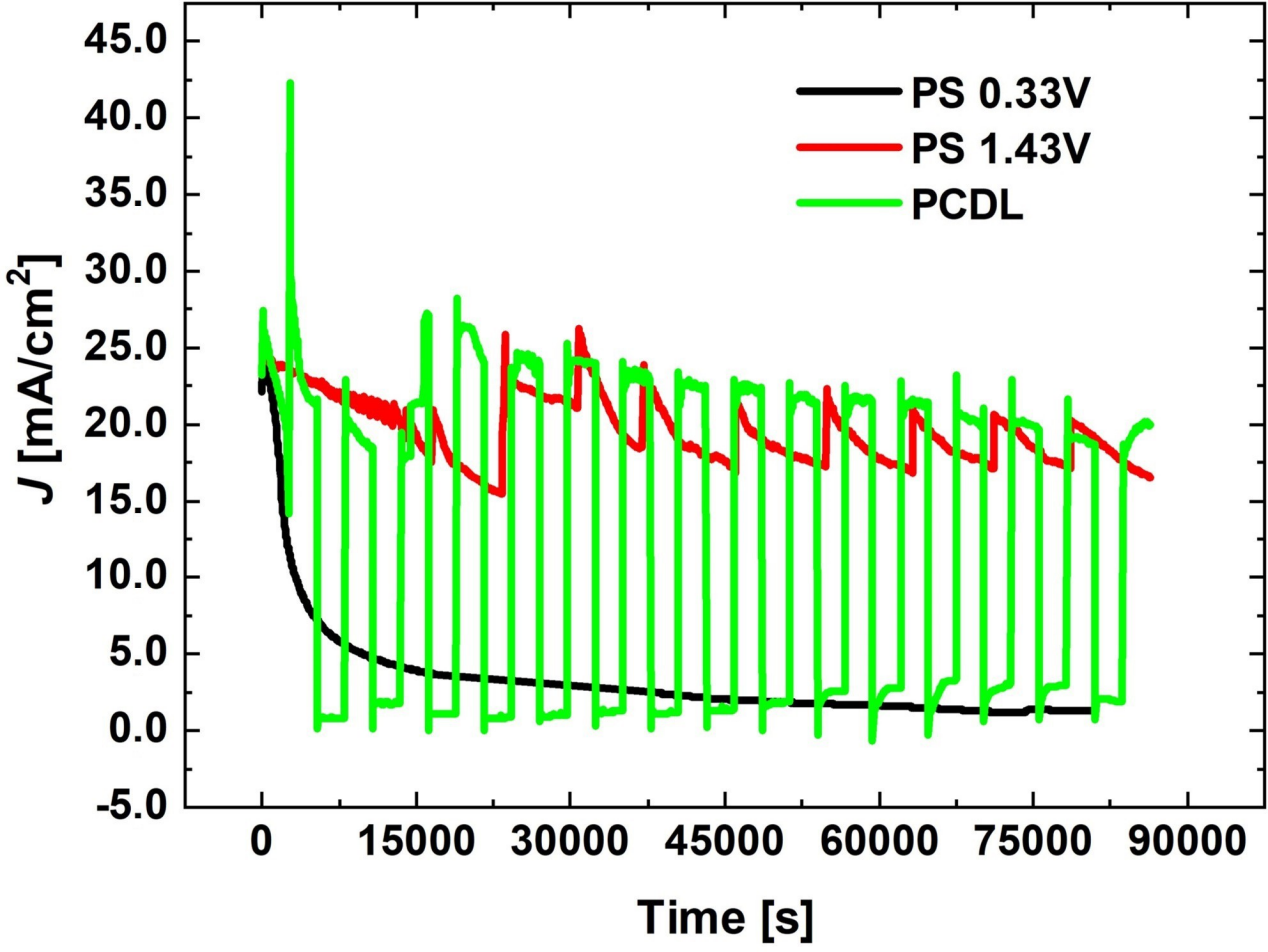
Comparison of potentostatic and PCDL controlled corrosion tests for 12 %Co‐WC HM grade.

In this work we have extended and tuned this approach to the more industrially relevant case of HM grades with unalloyed Co binder. Controlled corrosion tests were carried out with the PCDL method, switching between the two potentials identified in Section 2.1, as well as in the potentostatic mode at each of these two potentials: the results are compared in Figure 2.

The c.d. levels attained in the continuous and pulsed potential application modes are comparable. Nevertheless, it is worth noting that the anodic c.d. in the E_critic,PP_ period tends to grow during the PCDL test. This shows stable activity – due to the renewed exposure of a fresh binder‐rich surface – combined with an increase of electrode surface area, due to the progressive erosion of the disk edges. Instead, in the PS mode, c.d. decreases, witnessing pseudopassivation. Moreover, the PS c.d. trace at E_critic,OER_ potential exhibits sustained oscillations, due to the formation and release of large bubbles in hanging‐meniscus configuration and detachment of partially corroded HM chunks (see Section 2.2.2). Differently, the pulsed‐potential case shows a more stable c.d. level, corresponding to localized OER at the pseudopassive film / residual HM interface, accompanied by the detachment of pseudopassive film layers.

It can thus be concluded that the PS process at low (0.33 V) and high (1.43 V) anodic potentials yields demolition rates of 49.4±10.6 and 172.0±17.9 mg day^−1^, respectively, while that of the PDCL is 188.8±39.7 mg day^−1^. Even though PS demolition at high anodic potential and PDCL give rise to comparable demolition rates, the demolition products are profoundly different.

In fact, it is worth noting that, in the case of highly corrosion‐resistant HM grades, demolition is only feasible with PDCL, and material removal occurs exclusively in the form of sequential disk detachment. Instead, with unalloyed grades, it is possible to detach material even with the PS approach, if a high enough anodic potential is applied. Nevertheless, the PS process yields lumps of material containing both residual binder and oxidized W, while the PDCL one generates disks of essentially pure WO_3_, also thanks to the local acidification of the anolyte. Since Co is completely released to the liquid phase, this allows the automatic separation of the metal values.

#### 2.2.2 Corrosion Product Analysis

The corrosion products obtained with the HM electrochemical demolition approach were analysed from the chemical and morphological viewpoints, in order to assess the nature of the films detached from the sample and of the hydroxides and basic salts precipitating in the catholyte.

**Figure 3:**
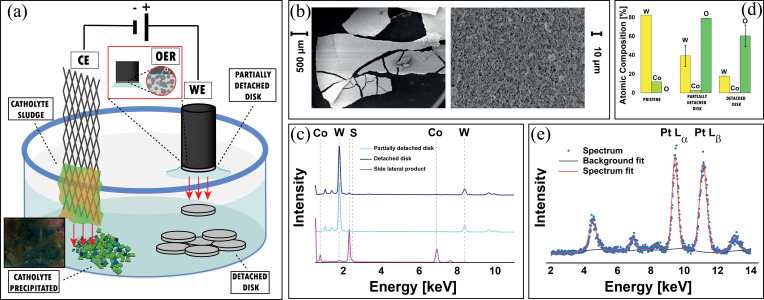
(a) Schematic of the formation and localization of demolition products in the cell. Inset: photograph of the cathodic precipitates. (b) SEM micrographs of detached corrosion product disks. (c) EDS spectra of: fully and partially detached corrosion product discs as well as of corrosion product layers forming on the side of the hardmetal cylinder. (d) Compositional analysis derived from the spectra of Panel (c). (e) Compositional analysis of the material grown onto the counter electrode (CE). Top panel: schematic of the CE regions analysed; two bottom panels, XRF spectra (blue plot: region in contact with the electrolyte, violet plot: region not exposed to the electrolyte).

PCDL demolition leads to the formation of new phases.: While it is proceeding, new compounds appear both at the sample surface and at the bottom of the cell (Figure 3a). Specifically: (i) detached disks dropped from the working electrode (WE) surface; (ii) partially detached disks adhering to WE surface; (iii) a corrosion product film, forming on the side of the WE, owing to wetting by capillary action and (iv) cathodic products, either precipitated in the catholyte or electrodeposited onto the CE surface.

The detached disks were collected by filtering them out from the electrolyte with glass‐fibre filter paper. Scanning electron microscopy (SEM) (Figure 3b) shows a morphology that is very similar to that found for hardmetal grades with alloyed binders,^[9]^ exhibiting the typical compact and micro‐cracked structure of electrochemically grown oxide film.^[21]^ The morphology of partially‐detached discs is identical to that of fully‐detached samples.

Energy Dispersive X‐ray Spectroscopy (EDS) data (Figure 3c) reveal that the fully detached disks contain only W and O and that no Co can be detected, in keeping with the findings of.^[9]^ Instead, partially detached corrosion product discs, show the presence of residual Co, possibly deriving from the substrate, owing to the limited thickness of the W oxide layer. Finally, the corrosion products forming on the side of the hanging‐meniscus electrode are strongly Co‐ enriched and contain S, suggesting that this material is essentially CoSO_4_ precipitated owing to supersaturation in the capillary film generated by the binder corrosion.

Based on thermodynamic considerations^[22]^ and colloid chemistry of aqueous Co(II) systems,[^4^, ^17^] the precipitate collected below the cathode is expected to consist of Co hydroxides with different degrees of hydration., iIn fact, the pH evolution of the initially neutral electrolyte leads to alkalinisation of the catholyte, as shown in Figure 1g. At the E_critic,PP_ and E_critic,OER_ potentials applied during PDCL, in the alkalinized electrolyte, Co(OH)_2_ and Co(OH)_3_ and CoOOH, the its dehydrated counterpart of the latter can form.[^22^, ^23^, ^24^] The respective characteristic colours (pink for Co(OH)_2_, brown for Co(OH)_3_ and blue for CoOOH,[^4^, ^24^, ^25^] see inset of Figure 3a), confirm that the cathodic precipitate is indeed a mixture of these species. The cathodically separated material was analysed by X‐ray fluorescence (XRF) (Figure 3e). The raw spectra were background‐subtracted by cubic spline interpolation, and fitted with linear combination of gaussian functions. Five well‐defined peaks are present, the ones of which at 4.50, 9.44, 11.10 and 12.94 keV, correspond to the Ti K and Pt L transitions, from the electrode material, while that at 6.90 keV is due to the Co K transition. Thus, XRF results prove the presence of pure Co, without W contaminations.

The compositional analyses described above thus prove that pure W oxides are confined in the corrosion product films that can be collected below the anode, while pure Co colloids can be separated from the catholyte and the cathode surface.

### 2.3 Sensitivity of the PCDL Process to Operating Parameters

**Figure 4:**
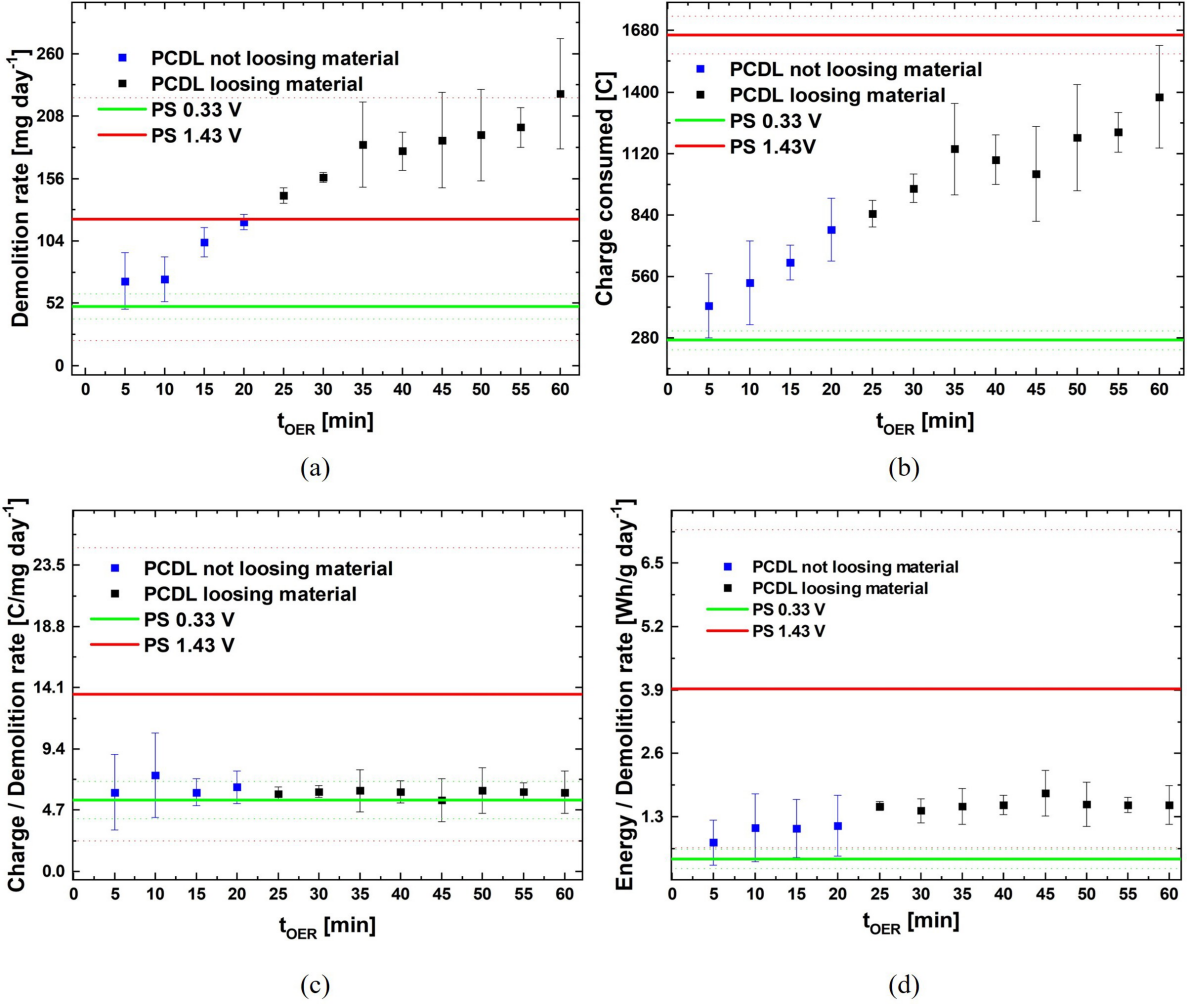
Efficiency indices as a function of the PCDL high‐potential step duration, compared to those for potentiostatic tests at the low (green lines) and high (red lines) potentials (mean values: continuous lines, sdev: dotted lines).

**Figure 5:**
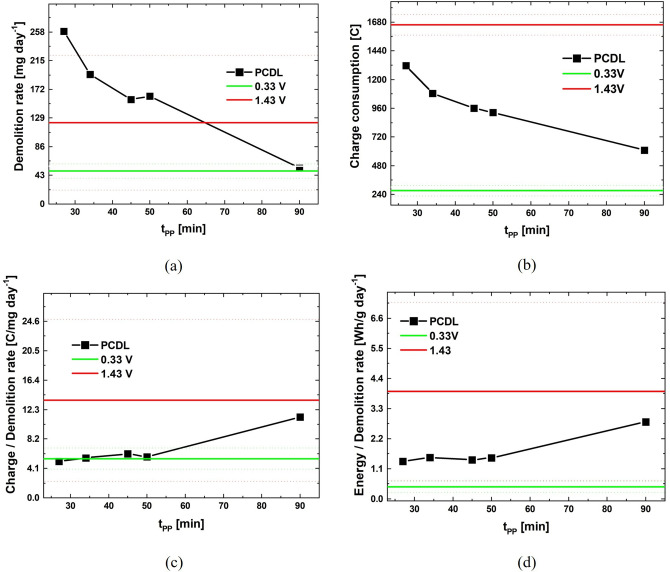
Efficiency indices as a function of the PCDL low‐potential step duration, compared to those for potentiostatic tests at the low (green lines) and high (red lines) potentials (mean values: continuous lines, sdev: dotted lines).

The PDCL process exhibits four operating parameters: E_critic,PP_, E_critic,OER_, t_PP_ and t_OER_. Setting appropriate values for them allows to fine‐tune the process performance, targeting demolition rate and energy consumption. The electrochemical materials science considerations reported in Section 2.1, allowed the definition of E_critic,PP_ and E_critic,OER_. As far as t_PP_ and t_OER_ are concerned, as reported in Section 2.2, we carried out experiments employing the values defined in [9] and optimized for HM grades with alloyed binders. This allowed to demonstrate an excellent performance of PDCL also for grades with Co‐rich binders. In the present section, we shall concentrate on the fine‐tuning of t_PP_ and t_OER_ for the materials considered in this study. Specifically, we have varied t_PP_ (keeping t_OER_=45 min) and t_OER_ (keeping t_PP_=30 min) in the ranges 27÷90 and 5÷60 min, respectively, and carried out tests lasting 24 h. In correspondence, we measured the demolition rate as well as the charge and energy consumed per unit time. For comparison with our PDCL data reported above and with literature results[^2^, ^8^] we also performed PS test for 24 h at E_critic,PP_ and E_critic,OER_. We recall that, as detailed in Section 3.2.1, the PS test at E_critic,PP_ does not lead to the detachment of fragments, but simply to Co^2+^ release into the electrolyte and that attack at E_critic,OER_ leads to the detachment of chunks of oxidized hardmetal containing both Co and W. The results of these experiments are reported in Figures 4 and 5.

From Figure 4a one notices that t_OER_ values in the range 5 to 20 min (blue squares) do not lead to measurable demolition, but just to the release of fine particles in the electrolyte. In these conditions, the c.d. levels attained in the E_critic,OER_ step are very low: this can be explained with the fact that the corrosion film properties do not exhibit the correct thickness and porosity to enable OER‐driven detachment. For t_OER_ values in the range 25–60 min, corrosion product discs are periodically released, in a way that is very similar to that described in [9] for grades with alloyed binder. The demolition rate positively correlates with t_OER_ and reaches values clearly above the levels attained with PS polarization, with the added value of Co/W separation. The charge consumed during the corrosion process and the ratio of this quantity to demolition rate are reported in Panels b and c of Figure 4. From these data, it is evident that PCDL has a high charge efficiency. On the one hand, this charge efficiency of PCDL is numerically, very close to that of the low‐voltage PS process, but, on the other hand, PS is not capable of yielding HM demolition and Co/W separation. Finally, Figure 4d reports the energy consumed rationed over demolition rate, showing that PCDL has a high energy efficiency, since the energy per mass corroded is only slightly higher than that of the ineffective low‐voltage PS process. In addition, from Figure 4, one can conclude that t_OER_ exhibits a vanishing impact on energy efficiency.

Figure 5a pinpoints that t_PP_ and demolition rate anticorrelate, showing that the properties of the pseudopassive layer are crucial for process efficiency. Moreover, the efficiency indicators of Panels b‐d prove that low t_PP_ values allow to reduce energy consumption: this is expected since the pseudopassivation process is already completed in correspondence of the lowest investigated values of t_PP_.

### 2.4 Electrochemical Demolition in the Context of the O.M.C.D. Tek Hub Hardmetal Recovery Model

**Figure 6:**
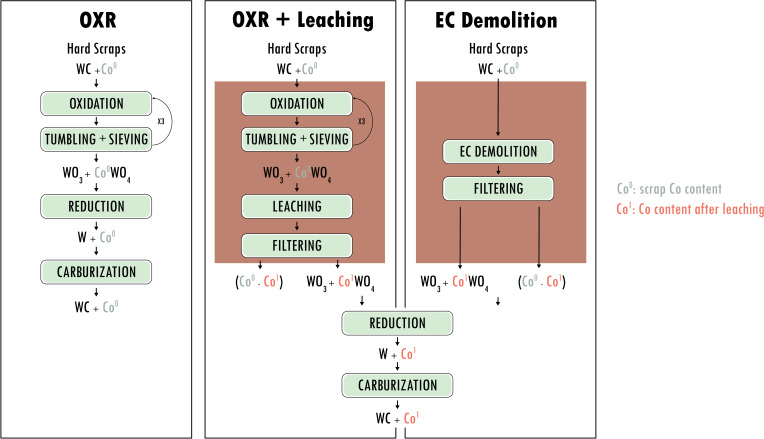
Flow‐diagram of the HM recovery processes of interest for O.M.C.D. Tek Hub. *Left column – OXR*: the existing pyrometallurgical process. At least three oxidation/tumbing+sieving cycles are required to complete HM demolition. *Central column – OXR+Leaching*: the OXR process with added leaching process, for the tuning of Co/W ratio: under development in the framework of the ReaQTool project. *Right column – EC Demolition*: the novel process, based on electrochemical demolition, presented in this work and funded by the MESCEL EU project. Unit operations and materials are coded with right‐angle and rounded‐corner boxes, respectively.

In this Section we describe the potential impact of the electrochemical demolition method proposed in this work on the whole HM recovery model of the industrial partner of this research. O.M.C.D. Tek Hub currently meets 60 % of its requirements for HM fabrication with internally recycled raw materials. Specifically, hard scrap is presently recovered with a semi‐direct pyrometallurgical process – denominated OXR (first column of Figure 6) –, based on oxidation, reduction and carburization. Though efficient, this approach – in addition to obvious environmental impact issues – exhibits the key technical drawback of keeping the same Co/W ratio in the recovered powder, as the scrap. This limits flexibility in reuse and mandates the use of virgin WC to tune the wide range of HM compositions produced. In order to impart flexibility to the Co/W ratio, O.M.C.D. is currently developing – in the framework of the ResQTool EU project – a novel OXR+Leaching process, which implements a leaching step after the thermal oxidation one (second column of Figure 6). Finally, the EC Demolition process (third column of Figure 6, funded by the MESCEL EU project) proposes to replace the thermal oxidation+leaching steps of the OXR+Leaching approach with a single step, running at room temperature on a neutral aqueous solution (red blocks in Figure 6). Table 2 reports the key technical characteristics of the three processes in terms of productivity, energy consumption and capability of separating Co and W. The OXR and OXR+Leaching processes still exhibit the highest productivity, but the novel PDCL electrochemical demolition approach has a demolition rate of a comparable order of magnitude. We emphasize the importance of this achievement, because PDCL allows to increase electrochemical demolition rate by over two orders of magnitude with respect to the literature state‐of‐the‐art, as detailed in [9]. In addition, PDCL compares favourably with OXR in terms of energy consumption. It is worth noting that the energetic cost of the merely electrokinetic part of the electrochemical process – as measured by the electrodic overvoltage (WE vs. RE) – is 2.9 kWh kg^−1^, importantly lower than the total energy consumption, that is strongly impacted by the ohmic drop of the cell. Thus, accurate electrochemical engineering of the demolition device that will be developed in the framework of the MESCEL project will strive to bring energy consumption as close as possible to the electrokinetic limit. Finally, it should be noted that only the OXR+Leaching and the EC demolition processes enable separation of Co and W: the latter methods implementing this capability with a one‐pot operation.

**Table 2:** Technical characteristics of the processes schematized in Figure 6. OXR is the pyrometallurgical process. PDCL and PS are the pulsed‐ and constant‐potential electrochemical processes, respectively.

Parameters	Unit	OXR	OXR+Leaching	EC demolition		
				PCDL	PS @ 0.33 V	PS @ 1.43 V
Energy consumption	kWh kg^−1^	6*	6.8^ǂ^	4.4*	0.8*	8.0*
Productivity	mg cm^−2^ h^−1^	30*	30^ǂ^	10*	2.6*	9.1*
Co/W separation		no	yes	yes	no	no

+: experimental data from real‐scale process at 800 °C. ǂ: estimates from ResQTool project. *: experimental data from laboratory‐scale process: cell voltage of 4 V.

## 3 Conclusions

This work extends the PCDL electrochemical approach for high‐rate HM demolition with concurrent physical separation of the Co‐ and W‐based corrosion products as the first step of the recycling process – pioneered by the Authors in [9] – to the single most industrially relevant case of grades with unalloyed Co binder. On the basis of electrochemical measurements, complemented with metallographic observations (SEM) and compositional analyses (EDS, XRF), we have defined the PCDL potential values between which the electrochemical control of the process is switched and the duration of the potentiostatic steps. Specifically, the active corrosion potential was identified to maximize cobalt extraction and formation of W‐based psudopassivating films that can be efficiently detached during the subsequent E_critic,OER_ high‐potential step. Moreover, in view of jointly optimizing the demolition rate and the energy efficiency, we have identified optimal hold times at the two potential values. Overall, optimizing the operating parameters E_critic,PP_, E_critic,OER_, t_PP_ and t_OER_, PCDL enables a productivity increase of a factor of more than 1.5 over the PS approach. This figure, in the case of HM grades with unalloyed binder, is not as high as with corrosion‐resistant grades, where an increase in demolition of ca. two orders of magnitude is obtained,^[8]^ but is it still of industrial interest, both *per se* and because it is accompanied by the added value of Co/W separation and decrease in energy consumption.

## 4 Materials and Methods

### 4.1 Preparation of HM Grades

HM samples were provided by F.I.L.M.S. S.p.A. of the OMCD Group. A typical commercial composition with 12 m% of Co and 2‐μm grain size WC is chosen. The samples were cylinders 9.88 mm in diameter and 33.5 mm in height, produced according to the standard Powder Metallurgy process. Extrafine Co powder supplied by Umicore (Olen, Belgium) and DS200 WC powder grade by H.C. Starck (Goslar, Germany) were used. The powders have been mixed in a lab‐size ball mill for 48 hrs, with a 3 : 1 ball:powders ratio, adding ethyl alcohol and a wax‐based pressing additive; at the end, they were dried under vacuum. Cylindrical samples were pressed at 120 MPa, dewaxed, presintered and sinterHIPped at 1400 °C under 50‐bar Ar pressure. Standard metallurgical quality control procedure was applied to characterize the sintered HM. The results are reported in Table 3. According to magnetic moment at saturation measurement the carbon content was in the expected C‐window. No unwanted phases or metallurgical defects were observed.

**Table 3:** Sintered HM characterization data.

Property	Unit	Value	Standard
Density	×10^3^ kg m^−3^	14.309	ISO 3369
Coercive Force	kA m^−1^	8.4	ISO 3326
Magnetic Moment at Saturation	%	90	ASTM B886
Vickers Hardness	HV30	1150	ISO 3878
Rockwell Hardness	HRA	88.3	ISO 3738
TRS	MPa	3484	ISO 3327
Microporosity	–	<A02B00C00	ISO 4499‐4
WC grain size	–	m	ISO 4499‐2

### 4.2 Electrochemical Measurements

Electrochemical measurements were performed in a three electrode electrochemical cell, with the working electrode (WE) in either hanging meniscus or immersed configurations, as specified where relevant. Platinized titanium expanded‐mesh was used as counter electrode (CE) (immersed area of 6 cm^2^). Ag/AgCl 3.5 M KCl (AMEL) was used as the reference electrode (RE). The cell construction ensured precise positioning of the three electrodes, ensuing high reproducibility of current density (c.d.) distribution and ohmic contributions to overvoltage. We employed a 0.2 M Na_2_SO_4_ aqueous solution, the rationale of this choice was to use a highly environmentally friendly and cheap solution, though at the cost of complex colloidal behavior and pH drifts, that we accurately monitored during the process. Moreover, acidification of the anolyte and alkalinization of the catholyte favour the sought‐after separation process.

Before each electrochemical measurement, all components were prepared with the following protocol. (i) The HM samples (WE) were weighted and washed in deionized water and acetone. (ii) RE, CE and glass cell components were immersed in clean aliquots of 10 % HNO_3_ solution for the removal of corrosion product traces. (iii) The RE was subsequently immersed in KCl solution and washed with Milli‐Q water. (iv) The CE was subsequently washed with Milli‐Q water and then sonicated (FALC Instruments ultrasound bath) for 5 min in isopropanol and for 5 min in Milli‐Q water.

A Versastat 3F potentiostat was employed. Following our previous work^[9]^ the electrochemical techniques adopted for this study were: (i) cyclic voltammetry (CV) sequences with progressive variation of the anodic terminal voltage (ATV) in steps of 0.1 V across the pseudopassive range and up to full‐developed transpassivity; (ii) potentiostatic measurements (PS) at fixed potential, with potential staircases (PSC) and PS square‐wave cycling.

Before CV measurements, the OCP was monitored for 30 min, to qual ify the surface conditions and to achieve a steady‐state, that was used as the cathodic terminal voltage (CTV). The scan rate was 1 mV s^−1^ and 10 cycles were acquired. With this approach we could precisely track the alterations in CV patterns caused by the gradual growth of irreversible processes associated with selective binder dealloying and initiation of WC attack. This allowed to identify the distinct potential ranges in which the different HM corrosion modes prevail, that we designed by assigning critical potential E_critic_ values. PSC tests were carried out to precisely assess the OER onset potential: 1 h PS periods were set, with 0.1 V steps. PS square‐wave cycling demolition loops (PCDL) consisted in a starting period 30 min at OCP, followed by a sequence of PS square waves of variable durations, switching between the E_critic_ value corresponding to full pseudopassivity (E_critic,PP_) and OER conditions (E_critic,OER_). Different durations of the PS steps at E_critic,PP_ and E_critic,OER_, t_PP_ and t_OER_, respectively, were considered in the tests described in Section 3.3. The demolition rate was measured at the end of the PS loop.

### 4.3 Microstructural and Compositional Characterization

SEM observations were made on a Zeiss Supra 40 under high vacuum.The electronic microscope is equipped with EDS elemental analysis device (Oxford Instruments). The oxygen content was measured by IR spectrometry with a LECO RO 400 instrument. X‐ray Fluorescence Spectrometry (XRF) was performed at the Nuclear Measurements Lab (NML) of the Politecnico di Milano, by using a primary beam of X‐ray, a sample positioning system and a Peltier‐cooled X‐ray detector based on a CdZnTe semiconductor crystal to detect the Co presence on the CE after the demolition process. The sample, tilted 22.5°, was irradiated with polychromatic X‐ray beam, while the detector was shielded by a Pb collimator with a 2 mm diameter hole with two purposes: selecting characteristics X‐rays and reducing background. The source‐to‐sample distance was set to 49 cm while the sample‐to‐detector one was set to 10 cm. CE was scanned in different zones by operating the X‐ray tube at 50 kV voltage and 1.06 mA current, in continuous mode. Each region was irradiated for a time exposure of 1000 s. For each peak appeared in the spectral analyses, the energy of the centroid was compared with the tabulated values of the characteristic X‐ray emission lines of all the elements, taken from the XRAYLIB library.^[26]^

## Conflict of Interests

The authors declare no conflict of interest.

5

## Data Availability

The data that support the findings of this study are available from the corresponding author upon reasonable request.
